# Supplementary material for: Adaptation of CD4 in gorillas and chimpanzees conveyed resistance to simian immunodeficiency viruses
Source: eLife. 2025 May 14;13:RP93316. doi: 10.7554/eLife.93316 (PMC12077880; doi:10.7554/eLife.93316)
Supplement: Figure 5—source data 2. [file elife-93316-fig5-data2.zip › figure 5 WB files uncropped labeled/figure 5 WB files uncropped labeled.docx]

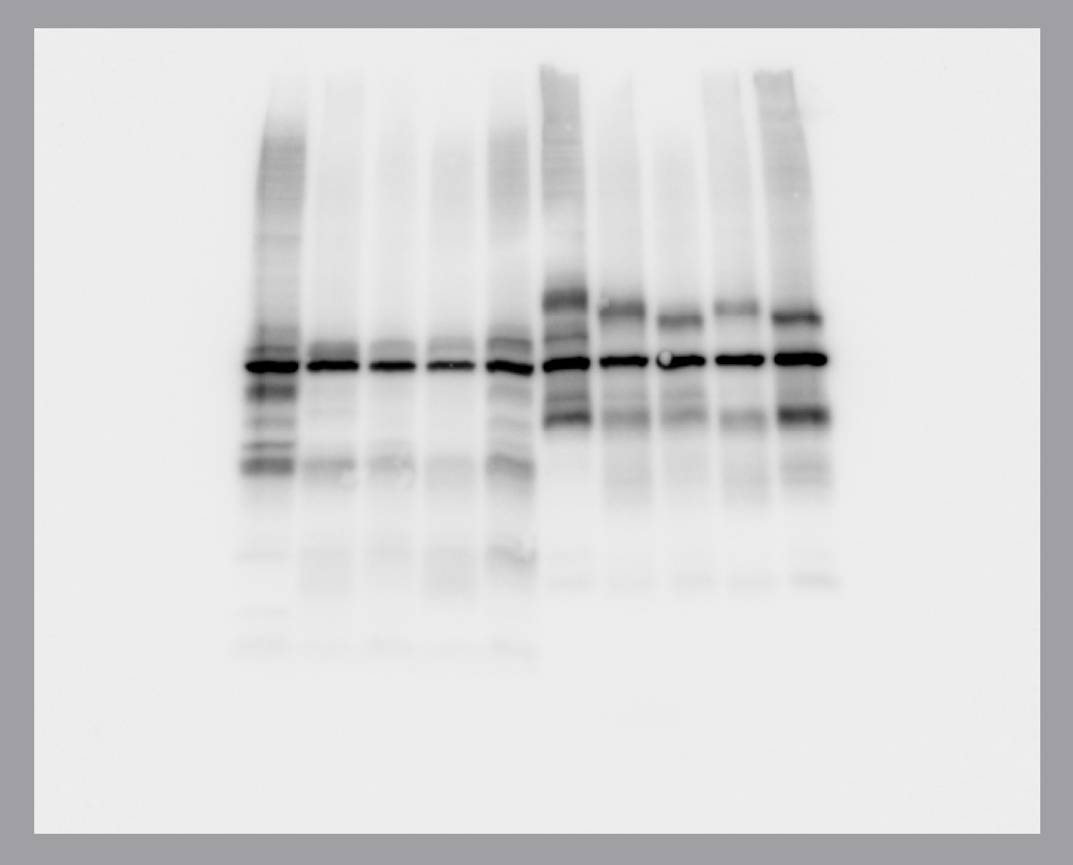

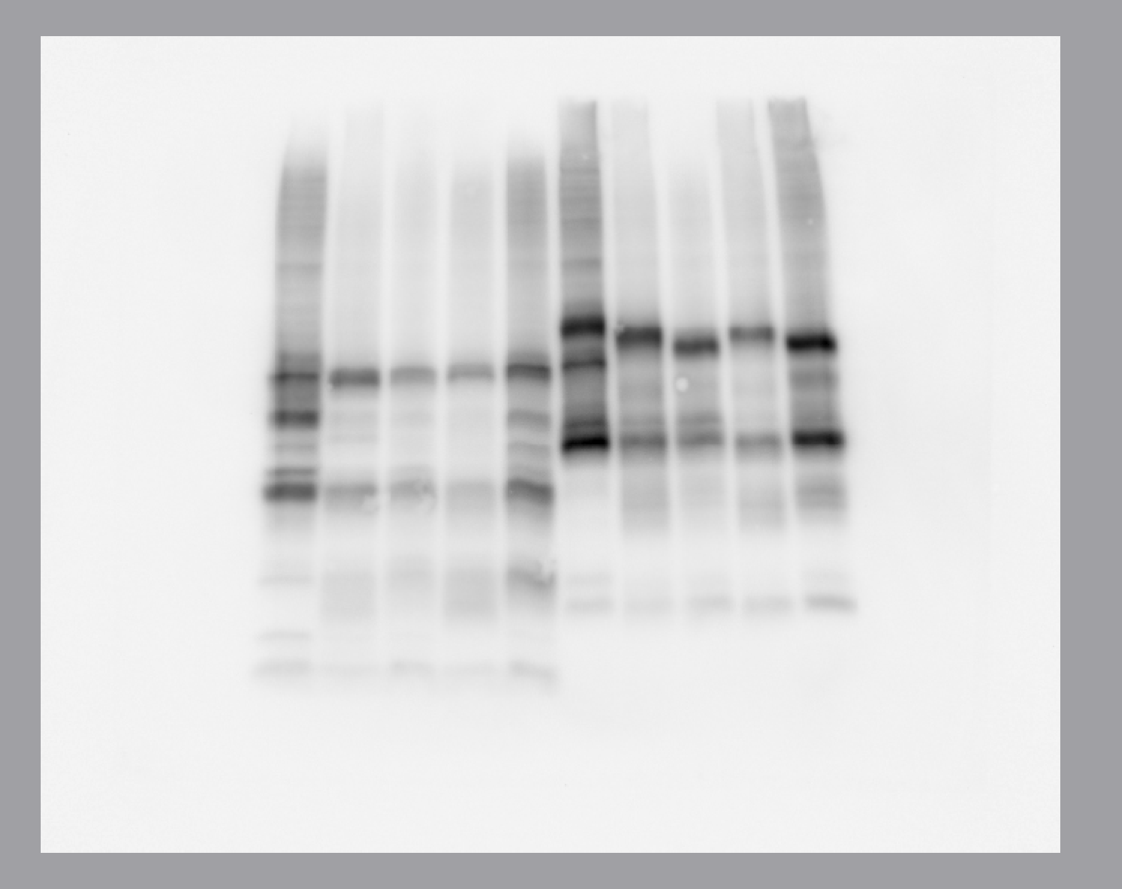


1

2

3

4

5

6

7

8

9

10

Blotted with anti-bActin antibody, using the same blot stained with anti CD4 antibody

1

2

3

4

5

6

7

8

9

10

Blotted with anti-CD4 antibody

NOTE: the raw images are inverted (left to right) compared to the final figure

1. no PNG, Human wt
2. no PNG, hum T15N
3. no PNG,gor 2
4. no PNG, gor 3
5. Chimp
6. Human wt
7. hum T15N
8. gor 2
9. gor 3
10. Chimp
