# Supplementary figures and images for: Adaptation of CD4 in gorillas and chimpanzees conveyed resistance to simian immunodeficiency viruses
Source: eLife. 2025 May 14;13:RP93316. doi: 10.7554/eLife.93316 (PMC12077880; doi:10.7554/eLife.93316)

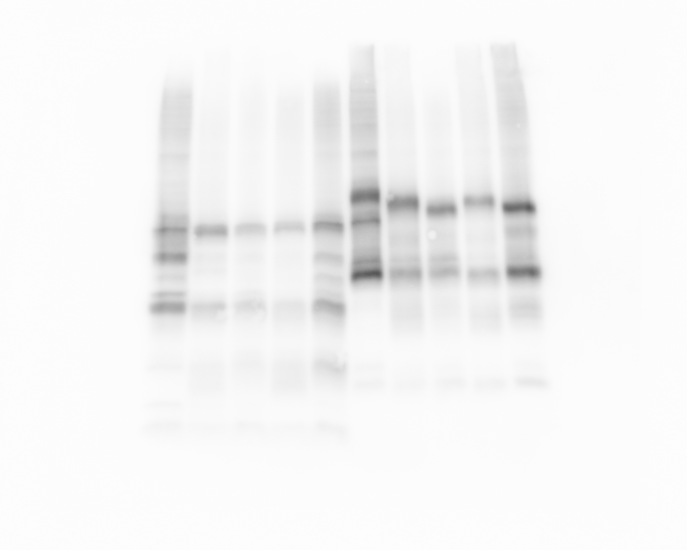

Supplement: Figure 5—source data 3. [file elife-93316-fig5-data3.zip › figure 5 WB files uncropped raw/CD4 raw.tif]

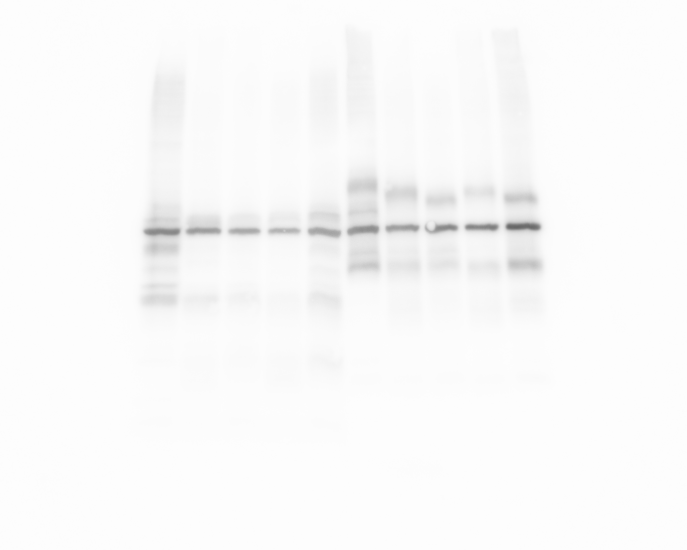

Supplement: Figure 5—source data 3. [file elife-93316-fig5-data3.zip › figure 5 WB files uncropped raw/bactin raw.tif]
